# Non-alcoholic fatty liver disease (NAFLD) is associated with dynamic changes in DNA hydroxymethylation

**DOI:** 10.1080/15592294.2019.1649527

**Published:** 2019-08-07

**Authors:** Marcus J. Lyall, John P. Thomson, Jessy Cartier, Raffaele Ottaviano, Timothy J. Kendall, Richard R. Meehan, Amanda J. Drake

**Affiliations:** a University/British Heart Foundation Centre for Cardiovascular Science, University of Edinburgh, The Queen’s Medical Research Institute, Edinburgh, UK; b MRC Human Genetics Unit at the Institute of Genetics and Molecular Medicine at the University of Edinburgh, Edinburgh, UK; c MRC Centre for Inflammation Research, University of Edinburgh, The Queen’s Medical Research Institute, Edinburgh, UK; d Division of Pathology, University of Edinburgh, The Queen’s Medical Research Institute, Edinburgh, UK

**Keywords:** Obesity, NAFLD, methylation, hydroxymethylation, steatosis

## Abstract

Non-alcoholic fatty liver disease (NAFLD) is now the commonest cause of liver disease in developed countries affecting 25–33% of the general population and up to 75% of those with obesity. Recent data suggest that alterations in DNA methylation may be related to NAFLD pathogenesis and progression and we have previously shown that dynamic changes in the cell lineage identifier 5-hydroxymethylcytosine (5hmC) may be important in the pathogenesis of liver disease. We used a model of diet-induced obesity, maintaining male mice on a high-fat diet (HFD) to generate hepatic steatosis. We profiled hepatic gene expression, global and locus-specific 5hmC and additionally investigated the effects of weight loss on the phenotype. HFD led to increased weight gain, fasting hyperglycaemia, glucose intolerance, insulin resistance and hepatic periportal macrovesicular steatosis. Diet-induced hepatic steatosis associated with reversible 5hmC changes at a discrete number of functionally important genes. We propose that 5hmC profiles are a useful signature of gene transcription and a marker of cell state in NAFLD and suggest that 5hmC profiles hold potential as a biomarker of abnormal liver physiology.

## Introduction

Non-alcoholic fatty liver disease (NAFLD) is now the commonest cause of liver disease in developed countries, affecting 25–33% of the general population and up to 75% of those with obesity [1–4]. NAFLD is strongly associated with obesity, insulin resistance, type 2 diabetes and cardiovascular disease [1,2], and the increasing prevalence of these disorders places a substantial burden on public health resources. Indeed, NAFLD is an early predictor of, and important determinant for, the development of type 2 diabetes and the metabolic syndrome [5]. NAFLD encompasses a spectrum of liver disease and whilst simple steatosis, involving hepatic lipid accumulation without inflammation or hepatocellular damage, is considered relatively benign, it can progress to non-alcoholic steatohepatitis (NASH), fibrosis and cirrhosis, and up to 27% of those with cirrhosis will develop hepatocellular carcinoma (HCC) [6,7]. The inter-individual variability in the risk of progression, coupled with a lack of understanding of underlying mechanisms has limited the development of effective biomarkers of risk and therapeutic interventions.

Recent data using both genome-wide and candidate gene analysis in human liver biopsy specimens have identified alterations in DNA methylation (5-methylcytosine, 5mC) over promoter and genic regions of functionally relevant genes and pathways in association with disease state, suggesting that epigenetic dysregulation may play a role in the pathogenesis and progression of NAFLD [8–10]. Further evidence comes from rodent studies showing that methyl donor restriction causes NAFLD-like liver injury [11] whilst supplementation protects against diet-induced hepatic steatosis [12]. DNA methylation (5-methylcytosine, 5mC) is important in the regulation of gene expression, and 5mC tends to be found over heterochromatic and repetitive portions of the genome. In contrast, 5-hydroxymethylcytosine (5hmC) is enriched over the bodies of expressed genes and regulatory enhancer elements, with differential enrichment across promoters in many tissues [13–17]. In adult liver, 5hmC is overrepresented in genes involved in active catabolic and metabolic processes [16]. Although 5hmC functions in DNA demethylation pathways, catalysed by the Ten-Eleven-Translocation enzymes (Tets) [18], it may also act as a functional DNA methylation mark [19]. Importantly, our appreciation of 5hmC patterns in disease is limited, since widely used bisulphite sequencing methods do not discriminate 5mC and 5hmC [20,21]. We have recently shown that ‘environmental’ insults can drive dynamic, reciprocal changes in 5mC/5hmC, suggesting that 5hmC profiles may be a useful dynamic signature of hepatic gene transcription and a marker of cell state [22]. For example, exposure to the non-genotoxic hepatic carcinogen phenobarbital results in altered 5mC and 5hmC profiles with a switch from a repressive to an active chromatin state at selected target genes [17], including the loss of 5hmC at a set of CpG island (CGI) promoters [23]. Furthermore, 5hmC patterns are dramatically altered in several human cancer types including HCC [23,24]. Thus, 5hmC profiles may be useful as a biomarker of both normal and abnormal liver physiology. Here we perform the first genome-wide analysis of liver 5hmC patterns in a mouse model of diet-induced obesity (DIO) and find that hepatic steatosis associates with reversible 5hmC changes at a discrete number of functionally important genes.

## Results

### DIO associates with hepatic steatosis and altered transcription of functionally relevant genes

**Figure 1.**
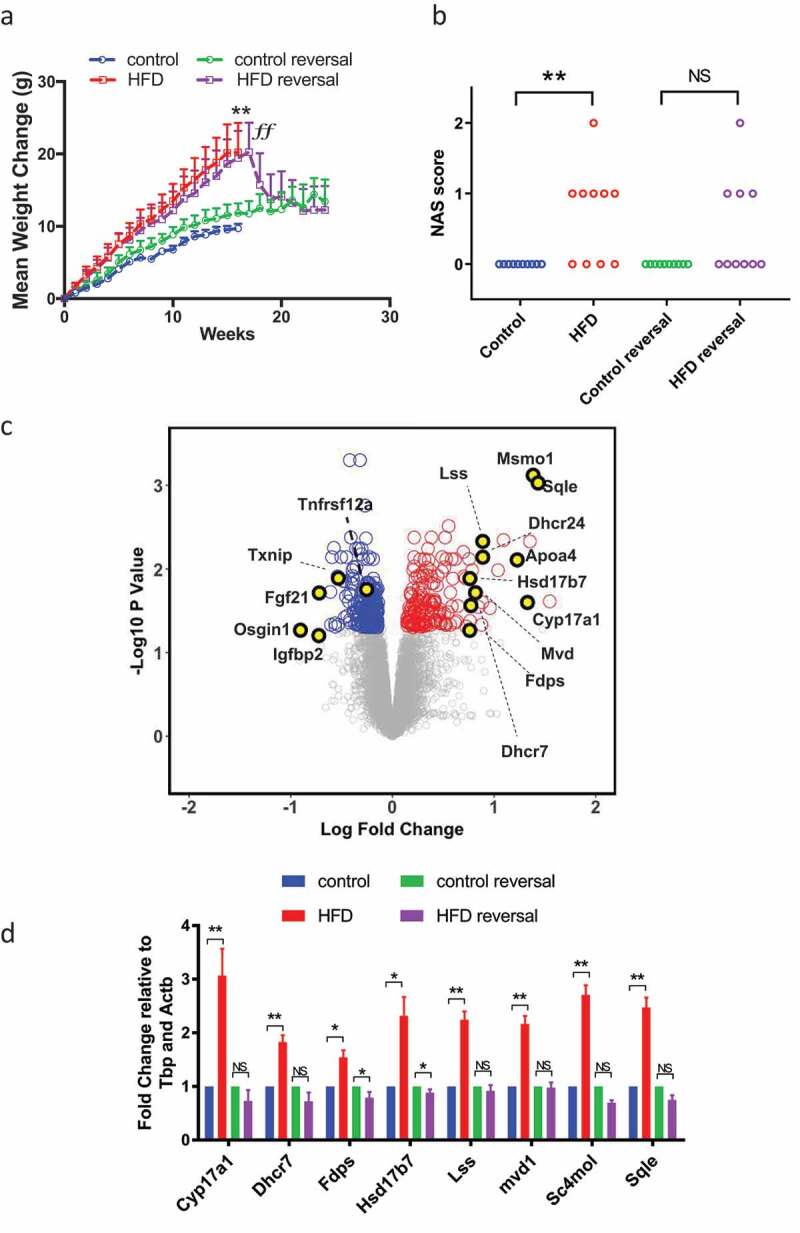
HFD associates with reversible hepatic steatosis and transcriptional derangement. (a) HFD induces weight gain. Data analysed by repeated measures two-way ANOVA with Tukey post hoc testing for multiple comparisons **p < 0.01 for HFD versus CON. (b) HFD was associated with an increase in hepatic steatosis score, assessed through the application of the NAS system. Data analysed by Kruskal Wallis test with Dunn’s post hoc test for multiple comparisons **p < 0.01 for HFD versus CON. (c) Volcano plot of CON and HFD liver showing up- (red) and downregulated (blue) transcripts (adjusted p < 0.05 (Benjamini-Hochberg) and change >10%). (d) qPCR of cholesterol synthesis mediators in CON, HFD, HFD-reversal and CON-reversal (n = 10/group) *p < 0.05, **p < 0.01 versus CON.

To explore the effects of diet-induced obesity on hepatic 5hmC we used a well-characterised mouse model of diet-induced obesity [25]. Maintaining adult male C57BL/6 mice on HFD or CON diets resulted in increased weight gain (Figure 1(a)) and increased fat pad weight (Supplementary Figure 1A), fasting hyperglycaemia, glucose intolerance (Supplementary Figure 1B) and insulin resistance (Supplementary Figure 1C). There were no differences in liver weight when corrected to total body weight, however HFD did induce hepatic periportal macrovesicular steatosis and a higher NAS steatosis score (Figure 1(b) and Supplementary Figure 2). No lobular necroinflammation or hepatocellular ballooning, the other features included in the NAS system, were present in the liver of any mouse in the study such that total NAS and the NAS steatosis score were always identical. As expected, given the lack of inflammation or ballooning to indicate steatohepatitis, there was no fibrosis in any mouse. We performed global hepatic transcriptome microarray analysis on CON (n = 4) and HFD (n = 8) mice. Analysis of the top 200 varying transcripts on microarray was sufficient to cluster individuals by Euclidean distance with minimal variance between individuals in each group (Supplementary Figure 3). At a threshold of 10% change with an adjusted p-value of 0.05, 154 genes were upregulated and 204 downregulated (Figure 1(c)). Such transcriptional derangement is comparable with human NAFLD datasets [9,26]. These included functionally relevant genes; the most enriched upregulated pathway was cholesterol biosynthesis GO:0006695 (adjusted P-value 8.2 × 10^−12^), with 12 of 154 genes directly involved in this process. Differential candidate gene expression was confirmed by qPCR (Figure 1(d)).

Weight loss results in improvements in the clinical picture of NAFLD and studies report resolution of NASH-associated changes in DNA methylation following bariatric surgery [9]. To examine any reversibility, 17-week HFD animals were switched to CON for 12 weeks (HFD-reversal) whilst controls were maintained on CON (control-reversal). HFD-reversal prompted rapid weight loss (Figure 1(a)), normalization of fat pads (Supplementary Figure 1A), glucose/insulin concentrations (Supplementary Figure 1B and C) and reduced hepatic steatosis (Figure 1(b) and Supplementary Figure 2) assessed by NAS steatosis scoring. Weight loss also resulted in almost complete resolution of gene expression changes (Figure 1(d)).

### Liver 5hmC profiling of control and HFD mice

**Figure 2.**
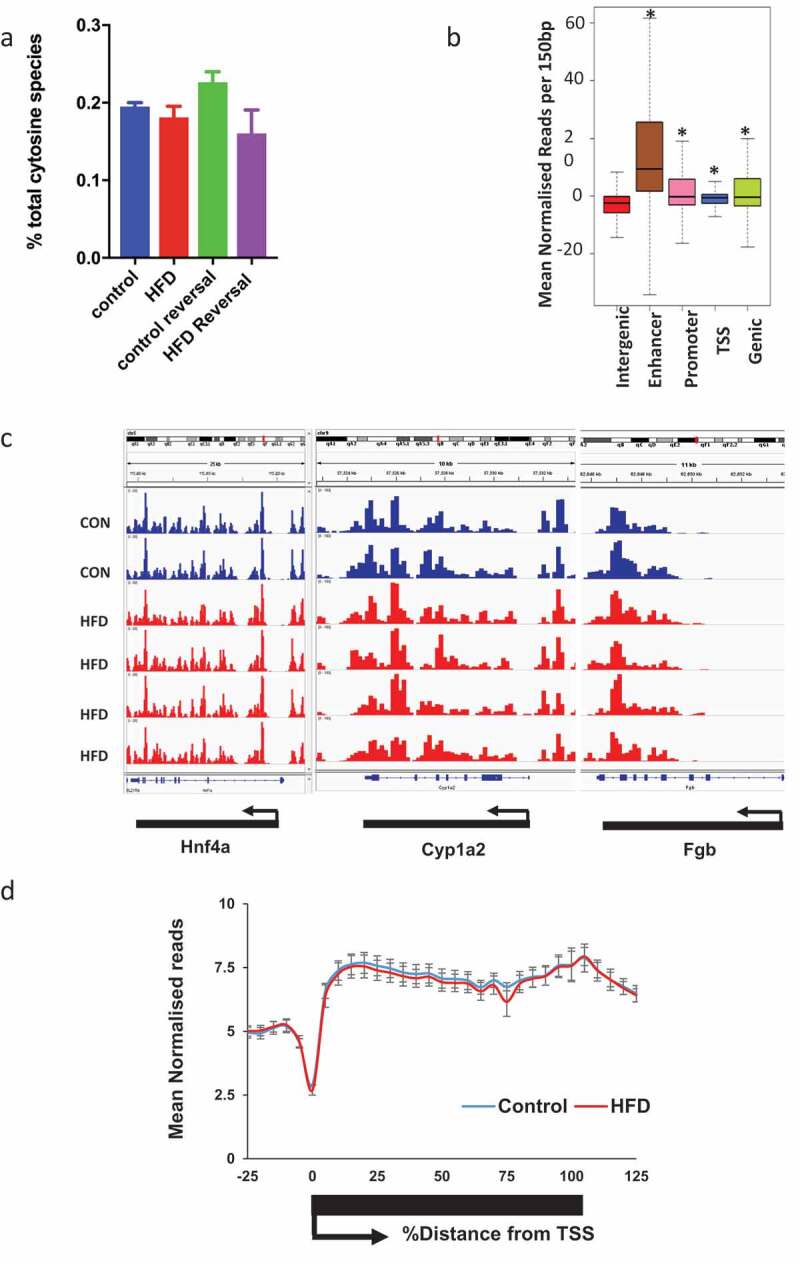
5hmC profiles are highly reproducible and HFD and weight loss do not associate with global 5hmC changes. (a) LC-MS analysis of hepatic 5hmC in CON and HFD mice. (b) Schematic representation of gene regions in relation to TSS. *p < 0.001 versus intergenic regions (Wilcox rank sum test). (c) Integrated genome viewer outputs of hMeDIP-seq experiments visualising 5hmC profiles over three constitutively active hepatic genes showing reproducibility between animals. Each histogram bar represents score over one 150 bp window. (d) Sliding window analysis of 5hmC profile over all genes in CON (blue n = 2) and HFD (red n = 4) mice.

**Figure 3.**
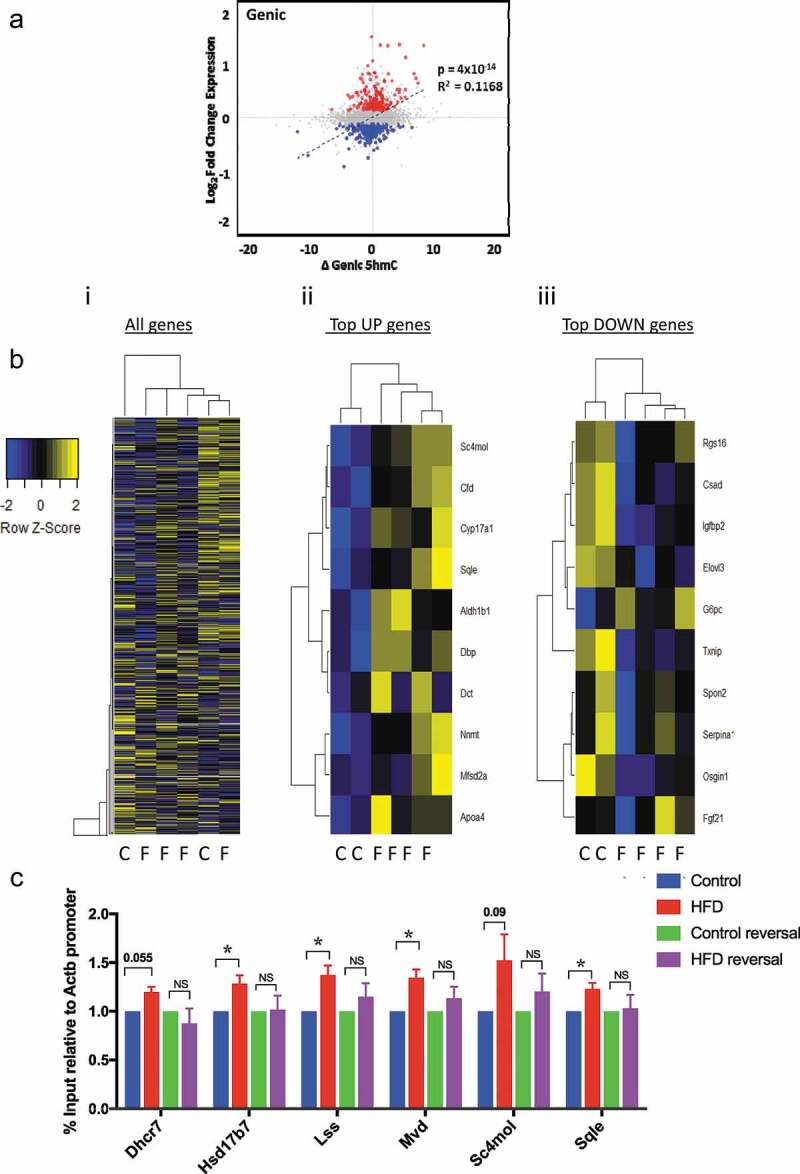
HFD associates with reversible genic 5hmC enrichment at discrete genes with biological relevance. (a) Scatter plot of changes in genic 5hmC versus transcriptional change; Δ5hmC = difference in mean number of normalised reads/150bp window. (b) Z-score heatmap analysis with hierarchical clustering based on average gene body 5hmC for (i) all genes (ii) top 10 induced and (iii) top 10 suppressed genes. (c) hmeDIP-qPCR validation of differentially hydroxymethylated loci and resolution in HFD-reversal group. Data are fold change versus CON analysed by one way Anova with Tukeys test for multiple comparisons * = p < 0.05.

Having established a model reflecting the metabolic and histological characteristics of NAFLD we proceeded to quantify total 5hmC by Liquid Chromatography–Mass Spectrometry (LC-MS). HFD was not associated with changes in global 5hmC levels (Figure 2(a)). To examine genome-wide 5hmC changes and investigate any discrete associations with transcriptional state, we undertook hydroxymethyl-DNA immunoprecipitation followed by semi-conductor sequencing (hMeDIP-seq) in CON (n = 2) and HFD (n = 4) liver. As previously reported [16,27], relative 5hmC enrichment was present in gene bodies and liver enhancer regions with lesser enrichment in promoters and minimal reads across transcriptional start sites (TSS) and intragenic regions (Figure 2(b)). Inter-individual 5hmC profiles were highly consistent (Figure 2(c,d)). Linear regression analysis comparing transcriptional and 5hmC changes revealed a highly significant relationship at gene bodies (Figure 3(a)), but not promoters, enhancers or TSS (Supplementary Figure 4). Although analysis of global 5hmC patterns did not differentiate CON from HFD (Figure 3(b)i), further analysis indicated genic 5hmC gain over a discrete number of HFD-induced (Figure 3(b)ii) and genic 5hmC loss at a separate distinct number of HFD-suppressed genes (Figure 3(b)iii) enabling clear stratification between control and HFD animals. 5hmC changes over functionally relevant transcripts were validated by hMeDiP-qPCR (Figure 3(c)). Weight loss resulted in reversal of 5hmC changes to control baseline (Figure 3(c)).

## Discussion

Observational human studies examining global DNA methylation dynamics across the NAFLD spectrum from hepatic steatosis to advanced NASH have shown aberrant 5mC patterns over both gene body and promoter regions of theoretically causative genes with an inverse correlation with gene expression and at least partial reversibility following bariatric surgery and resolution of liver injury [8,9,28]. Further evidence in support of a role for DNA methylation in NAFLD comes from studies in mice showing that dietary restriction of methyl donors or impairment of methyl donor metabolism causes liver injury similar to NAFLD [29,30] and dietary methyl donor supplementation appears to protect rodents from high fat/sucrose diet-induced hepatic steatosis [12]. However, since conventional bisulphite sequencing methods do not discriminate between 5mC and 5hmC [20,21], any role for 5hmC in the pathogenesis and progression of NAFLD is as yet uncharacterised. Using a genome-wide analysis of liver 5hmC in a mouse model of DIO we show that that hepatic steatosis associates with reversible 5hmC changes at a discrete number of functionally important genes.

Diet-induced hepatic steatosis is associated with altered expression of genes that play a role in NAFLD pathogenesis, in association with altered 5hmC profiles. We have previously shown that short- and long-term exposure to the non-genotoxic carcinogen phenobarbital leads to dynamic changes in the liver 5-hydroxymethylome [17,23]. Here we extend this work to show that disease-associated ‘environmental’ insults such as DIO can drive subtle changes in hepatic 5hmC, which may potentially be magnified in animals that progress to NASH and subsequent HCC [23]. Although there were no global differences in 5hmC, changes in 5hmC were identified at specific genes known to play a role in NAFLD pathogenesis, with 5hmC gain over a discrete number of HFD-induced genes and 5hmC loss at HFD-suppressed genes. This is consistent with human studies which show that 5hmC is enriched in genes with intermediate/high expression, and is overrepresented in genes involved in active metabolic and catabolic processes [16]. Additionally, the data are consistent with our previous studies using phenobarbital which show that 5hmC patterns are perturbed over a set of genes that are induced on phenobarbital exposure and that these are largely reproducible across multiple liver samples [17]. The observed alterations in the expression of genes that play a role in NAFLD pathogenesis, along with their altered 5hmC profiles are largely reversible with weight loss. This supports data from human studies reporting resolution of NASH-associated changes in DNA methylation with weight loss following bariatric surgery [9], and in mouse liver following drug exposure [14].

The progression from NAFLD, through NASH, fibrosis and cirrhosis is highly variable between individuals and is influenced by genes and environmental factors, with only a minority of individuals reaching end-stage liver disease and/or developing HCC [31]. This, coupled with a lack of understanding of the precise mechanisms underpinning the progression of NAFLD, makes prognosis and patient stratification difficult and has limited the development of reliable biomarkers of risk and effective therapeutic interventions. Recent work in mice and humans has identified methylation of the genic region of Igfbp2 as a ‘risk epigenotype’ in NAFLD and NASH [9,32]. In mice susceptible to the development of diet-induced obesity, Igfbp2 is hypermethylated and transcriptionally suppressed prior to the development of hepatic steatosis and this is stable over time [32]. Obese humans with glucose intolerance have increased DNA methylation at IGFBP2 in peripheral blood cells [32], and the IGFBP2 locus is hypermethylated in association with decreased expression in liver biopsy specimens from individuals with NASH [9]. Finally, overexpression of Igfbp2 reverses diabetes and steatosis in obese mice [33]. Our finding that DIO associates with decreased Igfbp2 expression (Figure 1(d)) and 5hmC loss (Figure 3(b)iii) builds on these data and supports the utility of this model for NAFLD studies.

Additional studies are required to determine the extent to which the 5hmC changes are functionally important or simply reflect changes in gene expression and whether they are a consequence rather than a cause of the disease. Importantly, these changes may be confounded by differences in cell composition in disease states. Nevertheless, our study has implications for the development of biomarkers, and further studies of 5hmC dynamics, for example, in *in vitro* models [34,35] and in humans will provide an exciting strategy to follow the molecular events associated with the progression of NAFLD and HCC. In terms of clinical utility, given the invasive nature of liver biopsies developing biomarkers in cell-free DNA (cfDNA) would be of clear benefit. Recent studies have shown the potential utility of 5mC analysis in circulating cell-free cfDNA in the stratification of liver disease subtypes [36] and the expansion of these studies to include 5hmC profiling may provide additional information, since for example, tumour-specific changes in 5hmC in cfDNA are detectable in a range of cancers including HCC [37]. Understanding the mechanisms underlying the changes in 5hmC and how 5hmC profiles relate to disease progression may additionally lead to the development of novel therapeutics to manage liver disease [38]. In conclusion, we propose that 5hmC profiles are a useful signature of gene transcription and a marker of cell state in NAFLD and suggest that 5hmC profiles hold potential as a biomarker of abnormal liver physiology in association with obesity and beyond.

## Materials and methods

### Animal care and husbandry

All experiments were carried out under a UK Home Office licence in accordance with the British Home Office Animals (Scientific Procedures) Act 1986, following ARRIVE guidelines and with institutional ethical committee approval. For all experiments, a 12-h light cycle (07.00 h to 19.00 h) and 12-h dark cycle was implemented throughout and the temperature was maintained at 22°C ± 2°C. Adult C57BL/6J males (Charles River) were maintained under controlled conditions in social groups of five animals per cage. Mice were maintained on control (CON, 11% kcal from fat; D12328, Research Diets) or a high-fat diet (HFD, 58% kcal from fat; D12331 Research Diets) for 17 weeks to induce obesity (n = 10/group). Glucose tolerance tests were performed at 17 weeks. After this, mice were killed at 17 weeks and liver and fat pads dissected, weighted and snap frozen on dry ice. To identify whether any changes were reversible with weight loss, we performed a second experiment in a further cohort of mice. In this ‘reversal’ experiment, a cohort of adult male C57BL/6J mice was maintained on identical diets (control diet D12328 or HFD D12331; n = 10/group) for 17 weeks. After 17 weeks, the control group was maintained on diet D12328 (control-reversal) whilst mice on the HFD were switched to the control diet (HFD-reversal). Both groups remained on the control diet for a further 12 weeks. Glucose tolerance tests were performed at 28 weeks. ‘Reversal’ group mice were killed at 29 weeks and liver and fat pads dissected, weighted and snap frozen on dry ice. A schematic diagram describing the experiment is presented in Supplementary Figure 5.

### Phenotyping

Mice were fasted for 6 h prior to intraperitoneal injection with 2 g glucose/kg body weight of 40% w/v solution D-glucose (Sigma). Blood glucose concentrations were quantified using a glucometer (One Touch Ultra, Roche). Insulin concentrations were measured using the Mercodia Ultrasensitive Mouse Insulin ELISA kit (Mercodia). HOMA-IR was calculated using the formula HOMA-IR =  (fasting plasma insulin x fasting plasma glucose)/22.5 [39].

For histology, liver sections were fixed in formalin 10% solution for 24 h, transferred to 100% ethanol and mounted in paraffin blocks prior to staining with haematoxylin and eosin or picosirius red. Stained sections were independently assessed by a consultant transplant liver histopathologist from the national liver transplant centre, experienced in evaluation of histology in clinical trials and translational models of NAFLD. The NAFLD activity score (NAS) [40] can be used to quantify features of NAFLD in human disease. The same features can be evaluated in rodent models of NAFLD, as previously described [41]. Features of steatotic injury were scored on a scale from 0 to 8 (steatosis 0–3, lobular inflammation 0–3 and hepatocyte ballooning 0–2) on H&E stained sections. Fibrosis was assessed from the PSR-stained sections by applying the fibrosis score used with NAS. Analysis was undertaken blinded to all other data.

### Transcriptome profiling

mRNA was extracted from snap frozen liver tissue and ‘on column’ DNase-treated using Qiazol, DNaseI and an RNeasy kit (Qiagen). 500 ng mRNA from CON (n = 4) and HFD (n = 8) were biotin labelled (Illumina TotalPrep, Life Technologies) and hybridised to Illumina Mouse WG-6 Arrays at the Wellcome Trust Clinical Research Facility Genetics Core, Western General Hospital, Edinburgh, UK. Intensity data were generated using a HiScan array scanner (Illumina) and analysed using iScan Illumina software. Raw data were uploaded to R version 3.12 (www.r-project.org). Quality control, background subtraction, stabilization of variance and RSN normalization were performed using the Lumi Package. Application of Bayesian statistical methods and differential expression with Benjamini-Hochberg adjustment for multiple comparisons was performed using the Limma Package (both packages from bioconductor (http://creativecommons.org/licenses/by/4.0/www.bioconductor.org). Plots were generated using Base R, Heatmap.2 (gplots package) or ggplot2. Unsupervised clustering was performed using Euclidean distance. For validation, mRNA was reverse transcribed using the High Capacity cDNA Reverse Transcriptase Kit (Life Technologies). Quantitative real-time PCR validation was performed using Roche Universal Probe Library (Roche) or Taqman PCR assays (Life Technologies) on the Roche Lightcycler 480 (Roche). Gene expression is presented relative to the mean of two housekeeping genes as indicated. Primers are listed in Supplementary Table 1.

### Global quantification of 5hmC using liquid chromatography–mass spectrometry

DNA extraction was performed using the Qiagen DNeasy Blood and Tissue Kit (Qiagen) and RNase treated with RNase A (Purelink, Ambion). Global quantification of 5hmC was performed using Liquid Chromatography–Mass Spectrometry (LC-MS). Nucleotide monophosphates were separated on 150 × 4.6 mm SeQuant ZIC-pHILIC column (Millipore (UK) Ltd) using a BioRS 3000 (ThermoFisher Scientific), with a gradient 90% to 5% B in 10 min, where B = acetonitrile and A = 20mM ammonium carbonate. Ions were analysed in negative mode using a Q-Exactive (ThermoFisher Scientific) with scan range 300–350 m/z and resolution 70k.

### Hydroxymethylcytosine DNA immunoprecipitation (hMeDIP)

DNA extraction was performed using the Qiagen DNeasy Blood and Tissue Kit (Qiagen) and RNase treated with RNase A (Purelink, Ambion). 20 μg genomic DNA was sonicated using a Bioruptor to fragments between 100 and 600 base pairs (mean 250–300 base pairs). 2.5 μg of sonicated DNA was diluted to 450 μl in TE buffer and denatured for 10 min at 90°C in a heat block before cooling for 5 min at 4°C and diluting to a final volume of 500 μl immunoprecipitation buffer (10 mM sodium phosphate (pH 7.0), 140 mM NaCl, 0.05% Triton X-100). Ten percent input was removed at this stage. One microliter of anti-5hmC antibody (Active Motif) antibody was then added to the remaining sample and incubated for 3 h at 4°C. 40 μl of Dynabeads protein G (Invitrogen) was prewashed with BSA 0.1% in PBS and added to the DNA/antibody mixture for 1 h at 4°C. Beads were then collected by magnetic rack, washed three times with 1 ml of cold IP buffer, re-suspended in 250 μl of digestion buffer (50 mM Tris-HCl pH 8.0, 10 mM EDTA pH 8.0, 0.5% SDS) and treated with 20 μl of proteinase K 20 mg/ml (Roche) in a thermoshaker at 1000 rpm, 55°C overnight. Beads were then removed using a magnetic rack and the enriched fraction and input samples were then purified using the Qiagen Qiaquik PCR Purification Kit (Qiagen) with elution in 22 μl of water. Adequate enrichment was determined using negative (β-actin and Gapdh promoter) and positive control regions (Tex19.1 promoter and H19 intragenic) as indicated in the primer list. 1:10 dilution was used for qPCR analysis. For semiconductor sequencing, 10 μl was amplified for 18 cycles using a SeqPlex DNA Amplification Kit (Sigma).

100 ng of DNA was used to generate a DNA library from each sample using the Ion Xpress Plus Fragment Library Kit (Thermo Fisher Scientific). During this process, DNA fragments were end repaired and ligated to specific barcode adaptors before being amplified (eight cycles) and twice purified using the Agencourt AMPure XP PCR Clean Up Kit (Beckman Coulter). Libraries were quality controlled using the Agilent Bioanalyser DNA HS Kit (Agilent) and pooled in equimolar pairs prior to template preparation using the Ion PI™ Hi-Q™ OT2 200 Kit (Thermo Fisher Scientific) and sequencing on the Ion Torrent semiconductor sequencer using the Ion PI™ Hi-Q™ Sequencing Kit (Thermo Fisher Scientific) and an Ion PI™ Chip Kit v3 (Thermo Fisher Scientific). For consistency, each sample was sequenced on a PI chip with its own input. Each sample was sequenced to a depth of ~30 million reads prior to quality control. Raw sequencing data were quality controlled, filtered and aligned using Ion Torrent suite software (Life Technologies) and then normalised to total reads in R using bespoke scripts. Relative 5hmC levels per 150 bp window were determined using the ‘sliding windows’ function on the Galaxy server at IGMM, Western General Hospital, UK. Genomic annotation data for mouse (mm9 build) analyses were downloaded from the University of California Santa Cruz Genome Bioinformatics Resource. Genomic co-coordinates of transcriptional start sites and stop sites were identified from the mm9 build of the mouse genome and downloaded from the University of California Santa Cruz research facility website (https://genome.ucsc.edu/). Promoter regions were defined as the region 1kb upstream of the TSS. Enhancer region co-ordinates were predicted by a chromatic signature of the presence of H3K4me1 but absence of H3K4me3 histone modifications and were downloaded from the mouse ENCODE project (http://chromosome.sdsc.edu/).

### Statistics

Prism GraphPad software (GraphPad Software Inc.) was used for statistical analysis of animal phenotype data. Data were routinely analysed for normality and outliers and non-parametric tests used where required. Data are shown as mean ± SEM.

### Data availability

Sequencing data have been uploaded to the Gene Expression Omnibus (GSE109138).
