# Supplementary material for: Non-alcoholic fatty liver disease (NAFLD) is associated with dynamic changes in DNA hydroxymethylation
Source: Epigenetics. 2019 Aug 7;15(1-2):61–71. doi: 10.1080/15592294.2019.1649527 (PMC6961686; doi:10.1080/15592294.2019.1649527)
Supplement: Supplemental Material [file kepi-15-1-2-1649527-s001.pptx]

## Slide 1
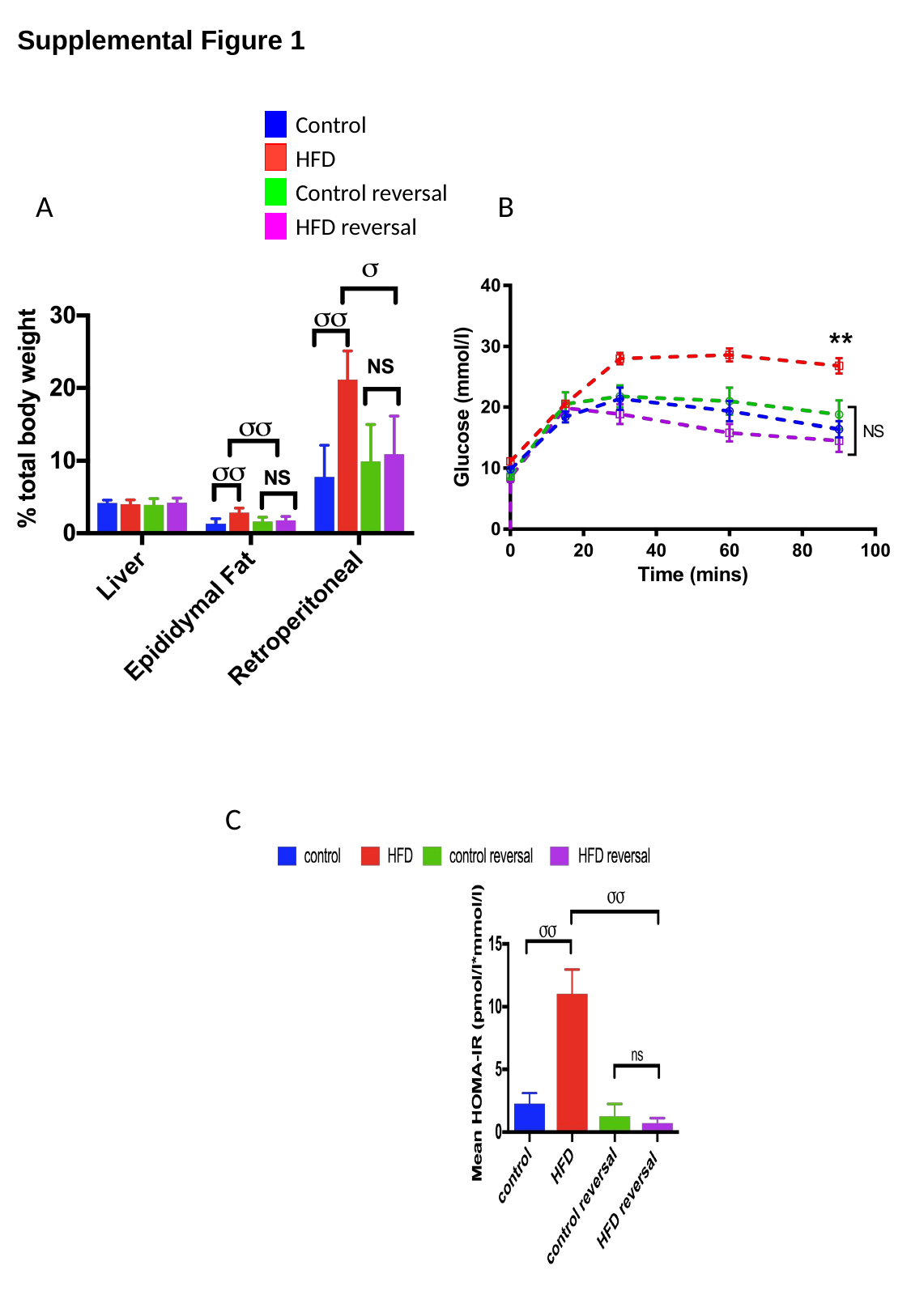

Supplemental Figure 1
Control
HFD
Control reversal
HFD reversal
A
B
C

## Slide 2
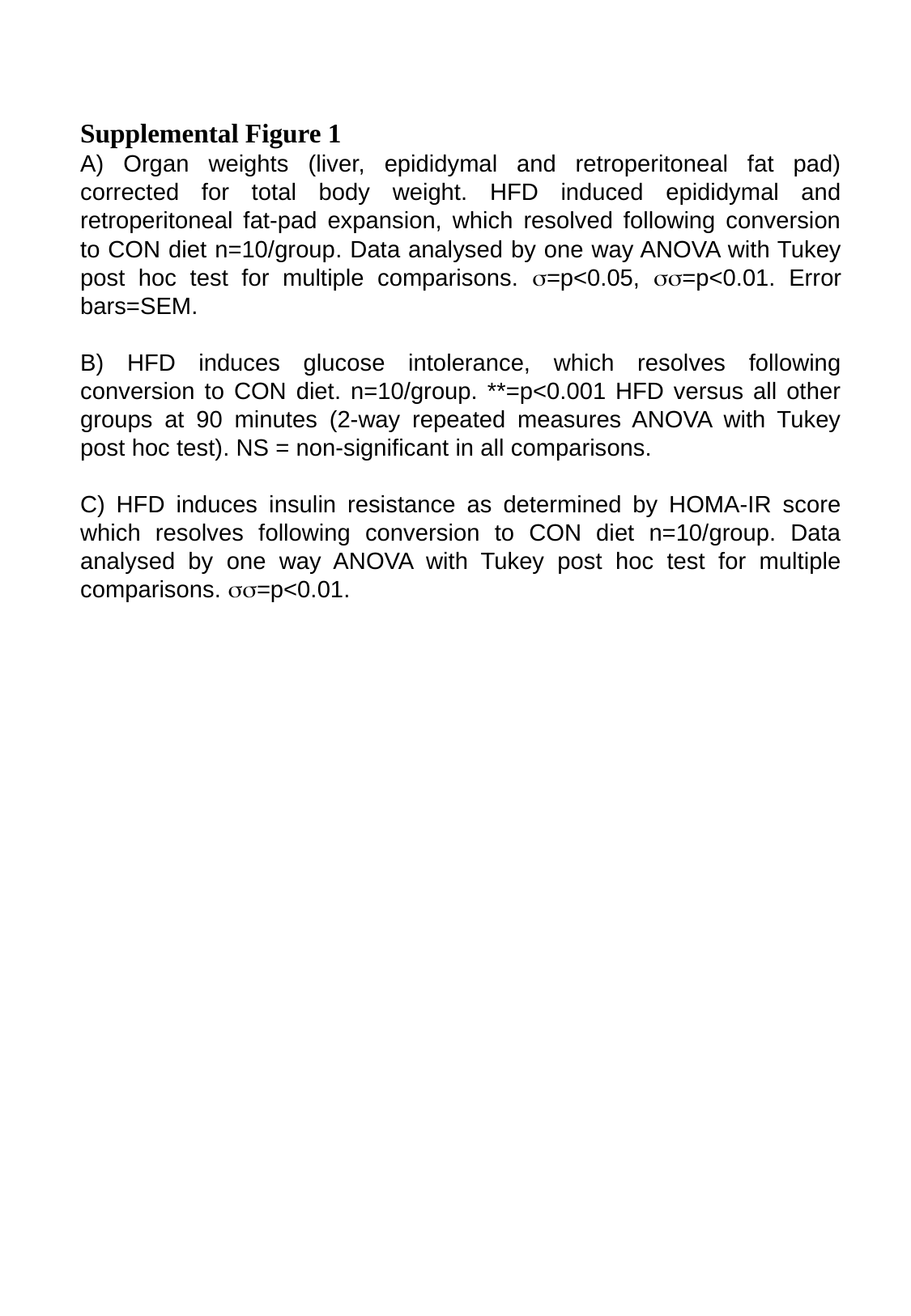

Supplemental Figure 1
A) Organ weights (liver, epididymal and retroperitoneal fat pad) corrected for total body weight. HFD induced epididymal and retroperitoneal fat-pad expansion, which resolved following conversion to CON diet n=10/group. Data analysed by one way ANOVA with Tukey post hoc test for multiple comparisons. =p<0.05, =p<0.01. Error bars=SEM.
B) HFD induces glucose intolerance, which resolves following conversion to CON diet. n=10/group. **=p<0.001 HFD versus all other groups at 90 minutes (2-way repeated measures ANOVA with Tukey post hoc test). NS = non-significant in all comparisons.
C) HFD induces insulin resistance as determined by HOMA-IR score which resolves following conversion to CON diet n=10/group. Data analysed by one way ANOVA with Tukey post hoc test for multiple comparisons. =p<0.01.

## Slide 3
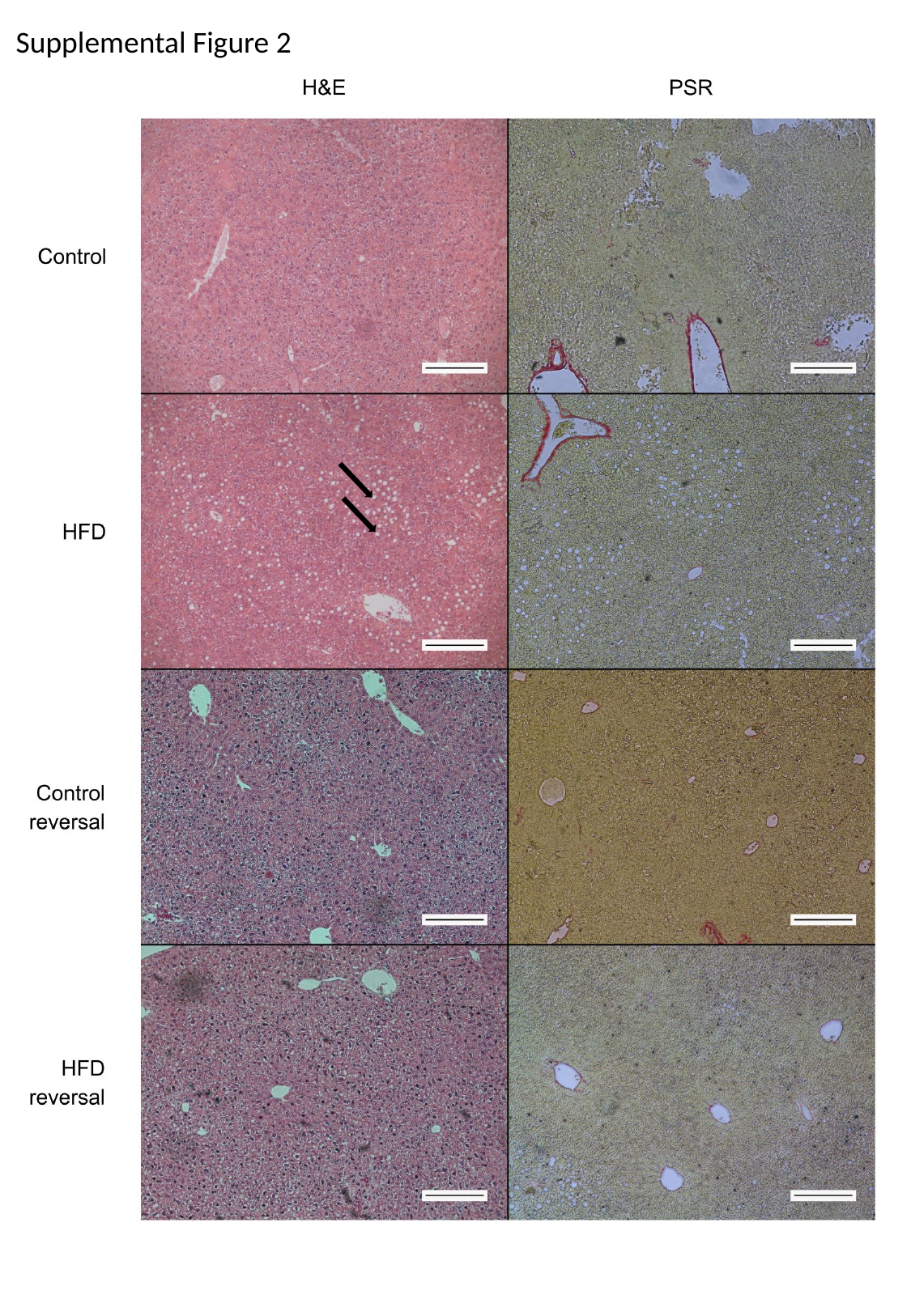

Supplemental Figure 2

## Slide 4
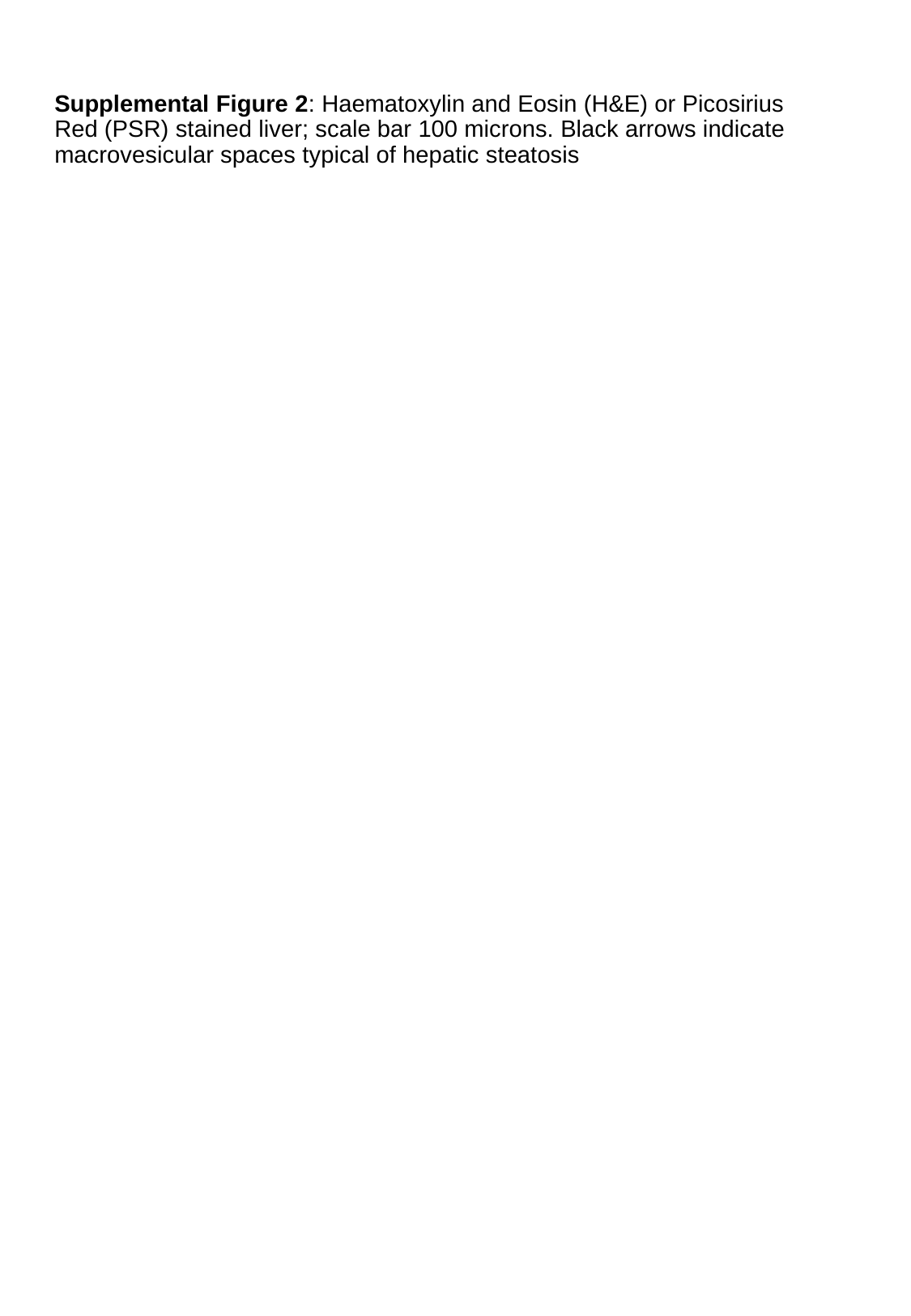

Supplemental Figure 2: Haematoxylin and Eosin (H&E) or Picosirius Red (PSR) stained liver; scale bar 100 microns. Black arrows indicate macrovesicular spaces typical of hepatic steatosis

## Slide 5
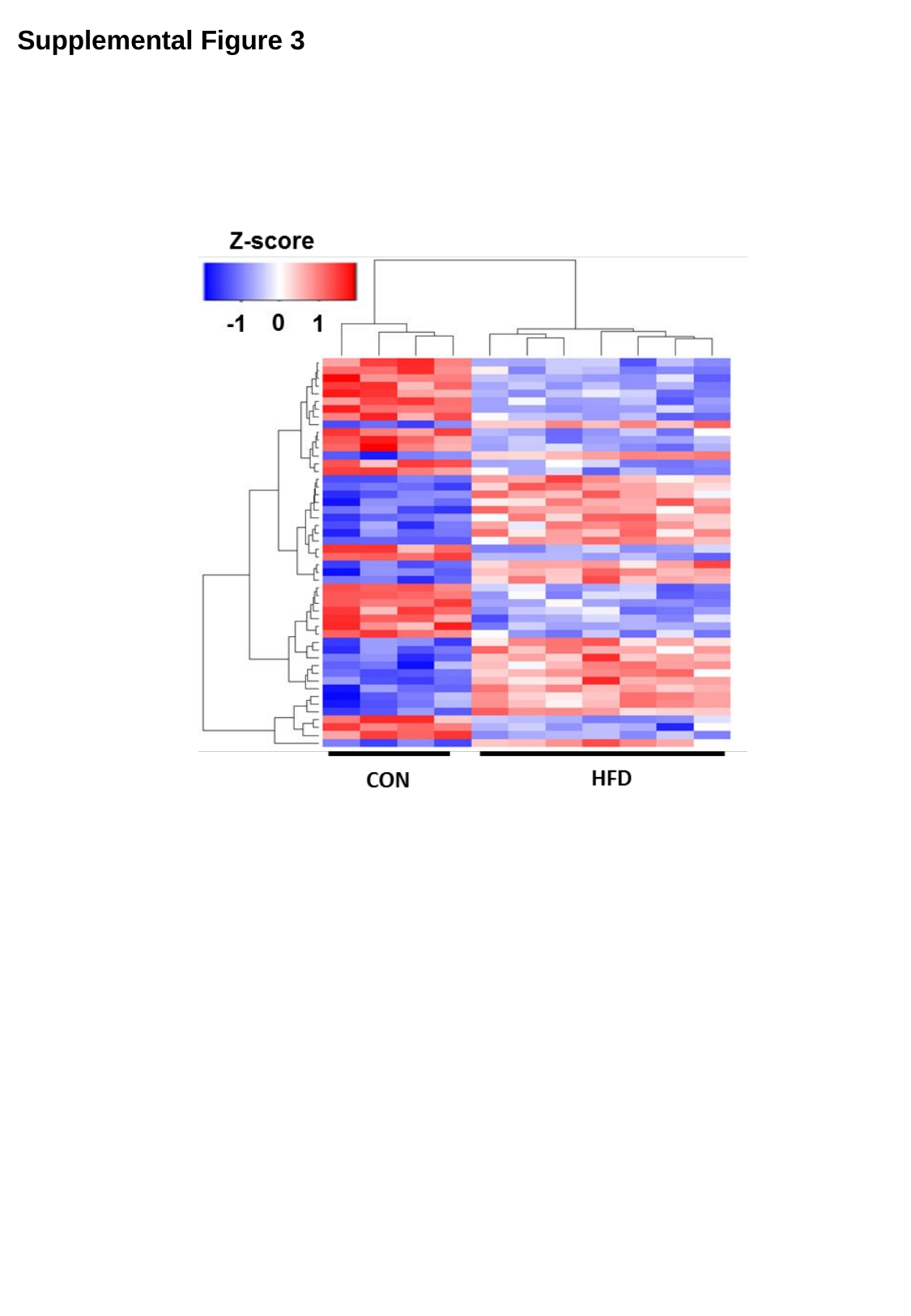

Supplemental Figure 3

## Slide 6
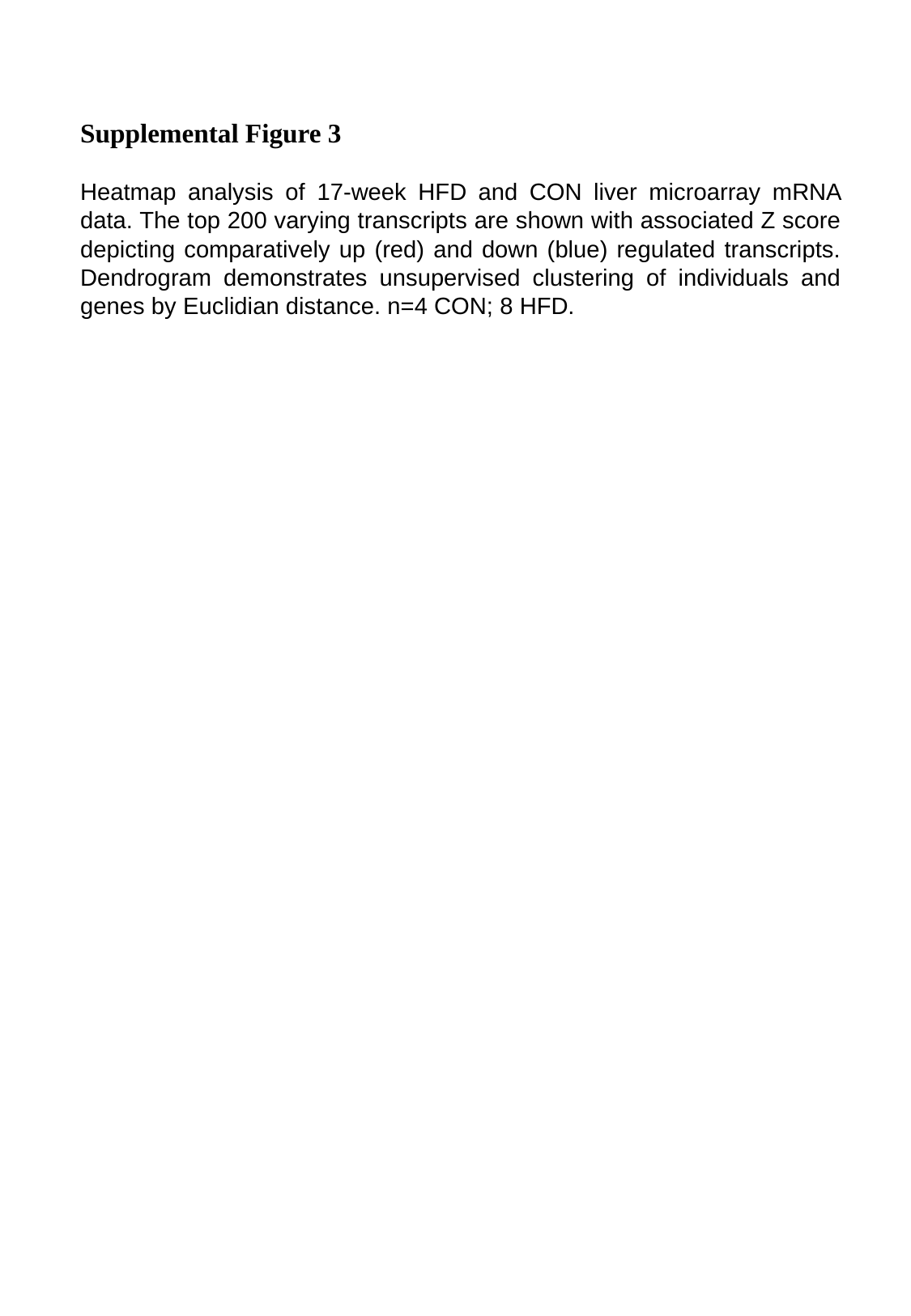

Supplemental Figure 3
Heatmap analysis of 17-week HFD and CON liver microarray mRNA data. The top 200 varying transcripts are shown with associated Z score depicting comparatively up (red) and down (blue) regulated transcripts. Dendrogram demonstrates unsupervised clustering of individuals and genes by Euclidian distance. n=4 CON; 8 HFD.

## Slide 7
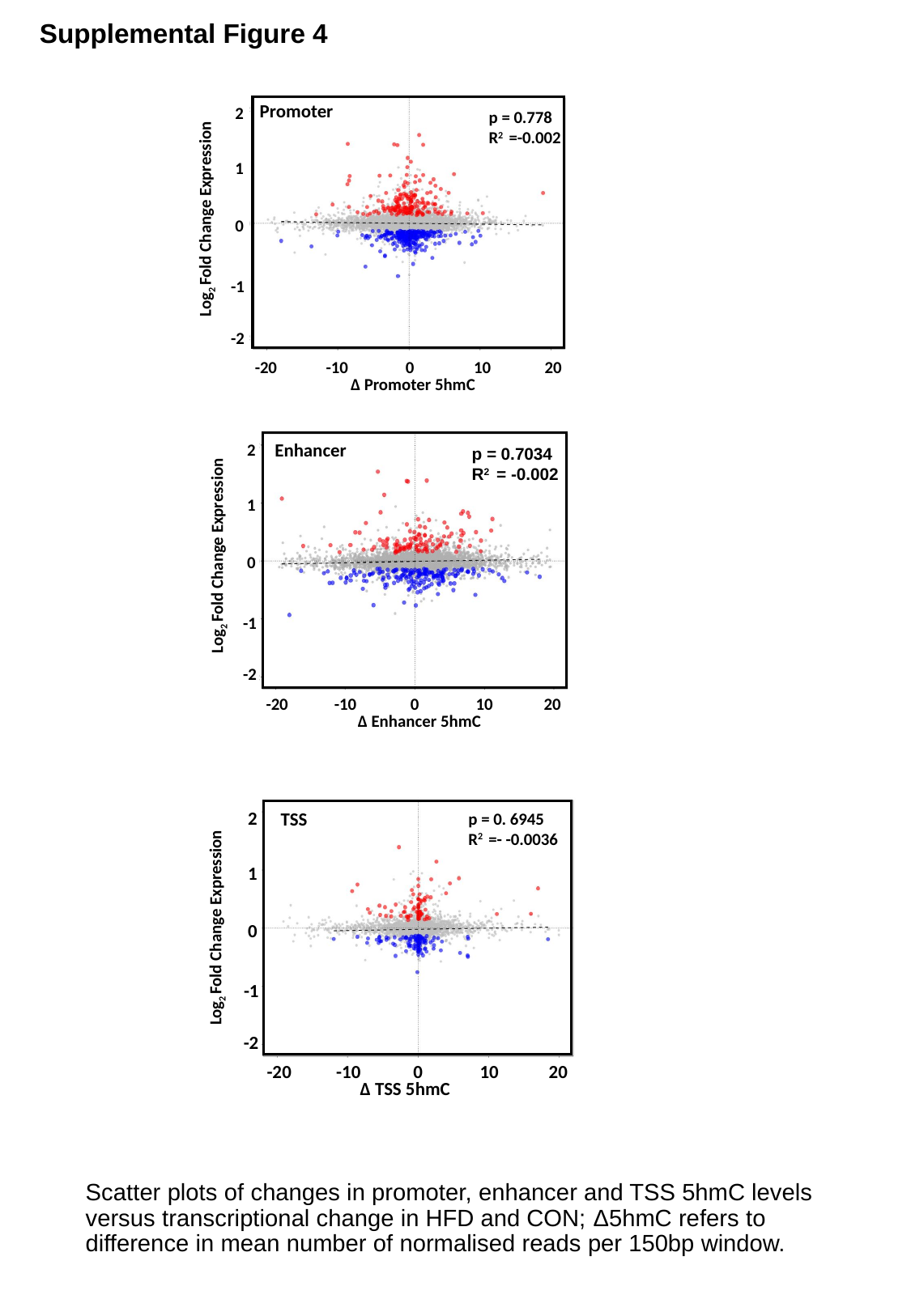

Supplemental Figure 4
2
1
0
-1
-2
-20
-10
0
10
20
Log2 Fold Change Expression
Δ Promoter 5hmC
p = 0.778
R2 =-0.002
Promoter
2
1
0
-1
-2
-20
-10
0
10
20
Log2 Fold Change Expression
Δ Enhancer 5hmC
p = 0.7034
R2 = -0.002
Enhancer
2
1
0
-1
-2
-20
-10
0
10
20
Log2 Fold Change Expression
Δ TSS 5hmC
TSS
p = 0. 6945
R2 =- -0.0036
Scatter plots of changes in promoter, enhancer and TSS 5hmC levels versus transcriptional change in HFD and CON; Δ5hmC refers to difference in mean number of normalised reads per 150bp window.

## Slide 8
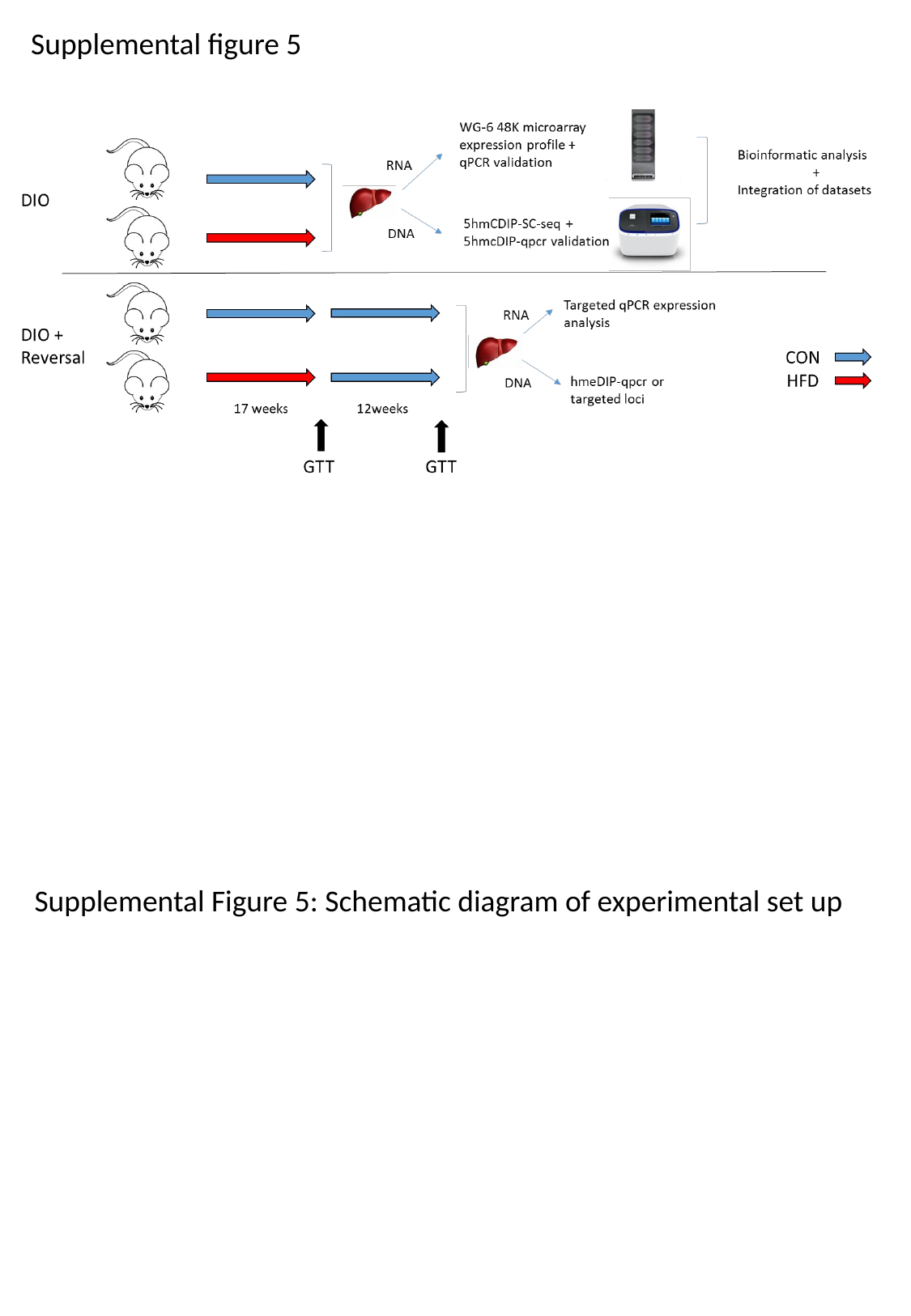

Supplemental figure 5
Supplemental Figure 5: Schematic diagram of experimental set up
