# Supplementary material for: Non-alcoholic fatty liver disease (NAFLD) is associated with dynamic changes in DNA hydroxymethylation
Source: Epigenetics. 2019 Aug 7;15(1-2):61–71. doi: 10.1080/15592294.2019.1649527 (PMC6961686; doi:10.1080/15592294.2019.1649527)
Supplement: Supplemental Material [file kepi-15-1-2-1649527-s002.docx]

**Supporting document.**

**Tet1 mediated DNA hydroxymethylation is important for the development of NAFLD**

Marcus J Lyall^1^, John P Thomson^2^, Jessy Cartier^1^, Raffaele Ottaviano^2^, Tim J Kendall^3^, Richard R Meehan^2^, Amanda J Drake^1^

1. University/British Heart Foundation Centre for Cardiovascular Science, University of Edinburgh, The Queen's Medical Research Institute, 47 Little France Crescent, Edinburgh EH16 4TJ, UK

2. MRC Human Genetics Unit at the Institute of Genetics and Molecular Medicine at the University of Edinburgh, Edinburgh, United Kingdom. EH4 2XU, UK

3. MRC Centre for Inflammation Research, University of Edinburgh, The Queen's Medical Research Institute, 47 Little France Crescent, Edinburgh EH16 4TJ, UK

**Supplemental Table 1: Primer lists for qPCR and hMeDIP-qPCR analysis**

| **Mouse Transcript** | **Forward** | **Reverse** | **UPL probe number** |
| --- | --- | --- | --- |
| Beta Actin | ctaaggccaaccgtgaaaag | accagaggcatacagggaca | 64 |
| Tbp | gggagaatcatggaccagaa | gatgggaattccaggagtca | 97 |
| Sc4mol | tcggaattgtgcttttgtgt | gcgggttgagaggaatatca | 2 |
| Lss | tgctaggtcccaggtccat | gagcagtgatgtcgggatg | 3 |
| Hsd17b7 | gtgcagatggatgtcagcag | aggcaggattccagcattc | 6 |
| Mvd | Ctgaatggtcgcgaggag | gagtgtccccgtcctctgt | 1 |
| Sqle | catgagtctccggaaagcag | Tgaagcacaacaccttctataaactt | 53 |
| Cyp17a1 | Life Technologies (Paisley, UK ) Taqman assay number Mm00484040_m1 | | |
| Dhcr7 | Life Technologies (Paisley, UK) Taqman assay number Mm00514571_m1 | | |

| **hMeDIP-qPCR** | **Forward** | **Reverse** |
| --- | --- | --- |
| Gapdh promoter | ccactccccttcccagtttc | cctataaatacggactgcagc |
| Actin Promoter | atgtacaggaatagcctccg | cttaagtgctcgatatccac |
| H19 Genic | gccaagagagaagaaggaga | gaatgttgaaggactgaggg |
| Tex19.1 Genic | gggagatatgtaaatgagctgg | catccttacctccctgactgag |
| Lss genic | acttgtcctcgatgtgccta | ggtagacttcattgccagtg |
| Sc4mol genic | taccccccacacatacaaac | acgtaattctcctgtgggca |
| Mvd genic | cattgaggtaggagatcggt | gagtctgttgtgcctgagcg |
| Sqle genic | cggttatggtggagagttta | tgtaacagaggagagcagga |
| Hsd17b7 genic | gagctgggcagagaaaaaca | caaatagaatcgcacagagg |
| Dhcr7 genic | cgttgctgcttttgtagatg | gagataattggggtgaagac |
